# Synthesis of Nano-Oxide Precipitates by Implantation of Ti, Y and O Ions in Fe-10%Cr: Towards an Understanding of Precipitation in Oxide Dispersion-Strengthened (ODS) Steels

**DOI:** 10.3390/ma15144857

**Published:** 2022-07-12

**Authors:** Stéphanie Jublot-Leclerc, Martin Owusu-Mensah, Vladimir A. Borodin, Joël Ribis, Ludovic Largeau, Ryan Schoell, Djamel Kaoumi, Marion Descoins, Dominique Mangelinck, Aurélie Gentils

**Affiliations:** 1 Université Paris-Saclay, CNRS/IN2P3, IJCLab, 91405 Orsay, France; owusumensah.martin@knust.edu.gh; 2 Department of Physics, Kwame Nkrumah University of Science and Technology, Kumasi GH233, Ghana; 3 NRC Kurchatov Institute, 123098 Moscow, Russia; v.borodine@mail.ru; 4 National Research Nuclear University MEPhI, 115409 Moscow, Russia; 5 Université Paris-Saclay, CEA, Service de Recherches Métallurgiques Appliquées, 91191 Gif-sur-Yvette, France; joel.ribis@cea.fr; 6 Université Paris-Saclay, CNRS, Centre de Nanosciences et de Nanotechnologies, 91120 Palaiseau, France; ludovic.largeau@c2n.upsaclay.fr; 7 Department of Nuclear Engineering, North Carolina State University, Raleigh, NC 27695, USA; rmschoe@sandia.gov (R.S.); dkaoumi@ncsu.edu (D.K.); 8 Aix-Marseille Université, CNRS, IM2NP, 13013 Marseille, France; marion.descoins@im2np.fr (M.D.); dominique.mangelinck@im2np.fr (D.M.)

**Keywords:** ion beam synthesis (IBS), ODS steels, ion implantation, FeCr, nano-oxide precipitates, precipitation, Y_2_O_3_, Cr_2_O_3_, Y_2_Ti_2_O_7_, core/shell structure

## Abstract

The properties of oxide dispersion-strengthened steels are highly dependent on the nature and size distribution of their constituting nano-oxide precipitates. A fine control of the processes of synthesis would enable the optimization of pertinent properties for use in various energy systems. This control, however, requires knowledge of the precise mechanisms of nucleation and growth of the nanoprecipitates, which are still a matter of debate. In the present study, nano-oxide precipitates were produced via the implantation of Y, Ti, and O ions in two different sequential orders in an Fe-10%Cr matrix that was subsequently thermally annealed. The results show that the oxides that precipitate are not necessarily favoured thermodynamically, but rather result from complex kinetics aspects related to the interaction between the implanted elements and induced defects. When Y is implanted first, the formation of nanoprecipitates with characteristics similar to those in conventionally produced ODS steels, especially with a core/shell structure, is evidenced. In contrast, when implantation starts with Ti, the precipitation of yttria during subsequent high-temperature annealing is totally suppressed, and corundum Cr_2_O_3_ precipitates instead. Moreover, the systematic involvement of {110} matrix planes in orientation relationships with the precipitates, independently of the precipitate nature, suggests matrix restriction effects on the early stages of precipitation.

## 1. Introduction

Oxide dispersion-strengthened (ODS) steels have attracted a great deal of attention as promising structural materials for next-generation fission and future fusion energy systems. Their excellent mechanical properties, specifically at elevated temperatures [1,2], are ensured by oxide nanoprecipitates whose many beneficial actions include the trapping of irradiation-induced defects, the suppression of bubble nucleation sites at grain boundaries by the provision of additional interfaces, and the inhibition of dislocation motion. ODS steels are usually produced via the mechanical alloying of powders, including Y_2_O_3_ and Fe-based powders, followed by consolidation processes at high temperature. The nature and size distribution of the resulting oxide nanoprecipitates highly depend on the many initial compositions, as well as on the many possible processing routes, with some consequential uncertainties regarding the identity of the precipitates. Many studies have focused on the investigation of the properties of these steels and their behaviour under irradiation [3,4,5,6,7,8], as well as on the proper description of the nanoprecipitate population [9,10,11,12,13]. No less important than understanding the behaviour of these steels is to understand the detailed mechanisms of formation of the nano-oxide precipitates, whose nature determines the thermal stability and radiation resistance of the material and whose size distribution influences its mechanical properties [14]. These mechanisms are, however, far from being understood in spite of experimental efforts carried out on conventionally produced ODS steels [15,16], as well as numerous theoretical studies [17,18,19,20]. In the present study, ion beam synthesis (IBS) was used to produce oxide nanoprecipitates in a model FeCr alloy with the purpose of obtaining key information about these mechanisms of formation. IBS is a nonequilibrium technique that consists of ion implantation at high fluence into a host material, generally followed by thermal annealing to induce the precipitation of encapsulated nanoparticles. It was, for example, successfully used to synthesize a wide range of compounds in silicon, such as GaAs [21] or CoSi_2_ nanocrystals [22], as well as metallic nanoparticles in SiO_2_ [23]. The ability to control several parameters—such as the impurity nature, its concentration beyond the solubility limit, the depth of implantation, and the temperature of implantation and subsequent thermal treatments—opens opportunities to disentangle the influence of various factors and to gain access to key elements in the nucleation and growth processes, in particular in the early precipitation stages that are difficult to capture. Successive dual implantations of Ti and O ions have previously been used in an attempt to produce Ti-rich oxide nanoprecipitates in FeCr [24,25]. Although Ti-oxides are thermodynamically favoured over Cr oxides, Ti did not appear to be involved in the precipitation at the early stages of nanoprecipitate formation, but instead was observed to be trapped in pre-synthesized Cr_2_O_3_ precipitates under thermal annealing from a temperature of 800 °C [25]. In the presently reported study, successive triple-ion implantations of Ti, Y, and O ions were performed into high-purity FeCr to move closer to the composition of ODS steels produced in the conventional way. In the conventional fabrication of ODS steels, all the alloying powders are introduced at the same time for the mechanical alloying process. The question of the influence of the order in the sequence of ion implantations thus arises. To address this point, two different sequential orders were considered. The present paper displays the results obtained after successive triple-ion implantations in these two different sequential orders: a striking effect is evidenced, and the formation of nanoprecipitates with characteristics close to those in conventionally produced ODS steels, in particular with a core/shell structure, is demonstrated for one of the sequential orders of ion implantation, namely, Y ➔ Ti ➔ O. 

## 2. Materials and Methods

### 2.1. Material

The material used in this study was a high purity Fe-10wt%Cr alloy fabricated at École Nationale Supérieure des Mines (Saint-Etienne, France). The nominal concentrations of C, N, O, S, and other impurities were lower than 0.001 wt% according to the supplier. The chromium content of 9.86 wt% was in the typical range for conventional ODS steels, usually between 9 and 14 at.%. The samples were provided as cylindrical rods of about 1 cm in diameter. They were cut into slices of about 1 mm in thickness. These slices were then mechanically polished with SiC abrasives down to a thickness of about 100 μm. Disks of 3 mm in diameter were then punched out of these slices before being electro-polished for a few seconds using a twin-jet electropolisher (Struers Tenupol-5) with 10% perchloric acid and 90% ethanol solution at −20 °C. During electropolishing, one surface was protected so that only one surface was mirror-polished. This electropolished surface was the surface that faced the beam during ion implantation and was easily recognizable for subsequent cross-section thin foil preparation. No heat treatment was performed before ion implantation.

### 2.2. Ion Implantation and Thermal Annealing Experiments

Triple ion implantations of Ti, Y, and O ions were performed successively into these 3 mm FeCr disks using the IRMA ion implanter of the JANNuS-SCALP facility at IJCLab [26,27]. Implantations were performed at room temperature (RT) to a fluence of 2 × 10^16^ cm^−2^ for Y and Ti ions, and to a fluence of 4 × 10^16^ cm^−2^ for O ions, to obtain approximately twice as much Y and Ti ions compared to O ions close to the mean projected range. To obtain a similar mean projected range between 40 and 50 nm for the three considered ions, implantation energies were chosen to be 170, 100, and 37 keV for Y, Ti, and O ions, respectively. The flux was approximately 7 × 10^12^ cm^−2^·s^−1^ for all implantations. Specimens were mounted at an angle of 7° with respect to the ion beam to avoid ion channelling effects. Using SRIM [28], the maximum ion concentration is calculated as roughly 9, 5, and 6 at.% for O, Ti, and Y ions, respectively. The peak vacancy concentration, occurring at 25–30 nm, amounts to 0.7, 2.7, and 5.1 vacancies per ion per Å. These calculations were performed in pure Fe using full damage cascades with a displacement energy of 40 eV [29]. The ion concentration and damage (dpa) profiles in the case of O and Ti implantations were reported in [24].

Two different orders in the sequence of implantation were considered: (i) Ti ➔ Y ➔ O and (ii) Y ➔ Ti ➔ O. Two sets of specimens, implanted with these two different sequences of implantation, were then annealed together for 2 h in the chamber of the IRMA ion implanter. Annealing was performed at 800 °C as well as 1100 °C, the latter temperature being close to the one at which consolidation is performed in the conventional fabrication process of ODS steels. The vacuum was in the order of 10^−7^ mbar throughout the annealing process. The annealing temperature was reached between 40 and 60 min, prior to the 2-h annealing.

### 2.3. Transmission Electron Microscopy Experiments

After ion implantation and subsequent annealing, a cross-section lamella of a few micrometres in length and width was extracted from the implanted surface using focused ion beam (FIB) at the Institut d’Électronique, de Microélectronique et de Nanotechnologie, IEMN (Lille, France). Prior to detailed compositional and structural characterizations, preliminary conventional and energy-filtered transmission electron microscopy (EFTEM) experiments were performed on the TECNAI G^2^ 20 Twin microscope of the JANNuS-SCALP facility, equipped with a Gatan GIF Tridiem apparatus.

Qualitative compositional analysis was performed using STEM-EDX with ChemiSTEM technology (4 detectors) at the Department of Materials Science and Engineering, North Carolina State University, Raleigh, NC, USA, on a FEI Talos FX200 G2, as well as on the probe-corrected FEI ThermoFisher Themis Titan 200 microscope of the C2N-PANAM platform, Palaiseau, France. The structural characterizations of the implanted FeCr matrix and of the synthesized nanoprecipitates were performed by either HRTEM on a JEOL 2010F FEG 200 kV at CEA-SRMA, Saclay, France, or by STEM-HAADF on the Themis Titan microscope of the PANAM platform cited above. Analysis of the HRTEM pictures was performed through analysing fast Fourier transforms (FFT): The distances and angles measured on the FFT patterns were compared with electron diffraction patterns in different zone axes, calculated using SingleCrystal software for oxides of different compositions and structures. In addition, simulation of the HRTEM pictures was performed using Pierre Stadelmann’s JEMS electron microscopy simulation software [30] on zone axes predetermined by the FFT analysis: The simulated pattern was properly oriented in plane with respect to the HRTEM picture by comparing the position of atoms calculated using JEMS and CrystalMaker software, as well as a proper orientation of corresponding SingleCrystal electron diffraction pattern (related to CrystalMaker atomic structure), as compared with the calculated FFT. All software used for crystallographic analysis was run using crystallographic structures files (.cifs) provided by The MaterialsProject [31] (many oxide structures were tested—the structure that best reproduced the FFT of Y_2_O_3_ precipitates, reported in Section 3.3, is the cubic structure with space group Ia3 described in the MaterialsProject file mp-2652). In a similar way, precipitates imaged using STEM-HAADF were analysed using FFT patterns. When possible, the calibration of distances in the FFT patterns was performed by identifying matrix orientation and distances either in the picture used for the precipitate analysis, or in a picture taken with the same magnification. As a last resort, the image calibration of the microscope was used. For the sake of simplicity, index j is not reported for hexagonal structures in the following.

### 2.4. Atom Probe Tomography Experiments

Atom probe tomography (APT) experiments were conducted at IM2NP Marseille using an atom probe LEAP 3000 XHR, Imago Scientific Instruments, equipped with a 532 nm wavelength laser. Experiments were performed at 80 K in the laser-pulsing mode with pulse width of 10 ps and an energy from 0.5 to 1.2 nJ. For these experiments, APT tips were extracted from ion-implanted and annealed electropolished disks with the FIB lift-out method using a FEI SEM Helios 600 Nano-Lab-focused ion beam. Three-dimensional reconstruction was performed using the IVAS software. 

## 3. Results

After room temperature (RT) implantation, no precipitates could be detected by conventional TEM. EFTEM elemental maps also showed no evidence of nanoprecipitate formation, in agreement with previous results obtained after the RT implantation of Ti and O ions [25,32]. In this previous study, as well as in an in situ annealing experiment [24,25], nanoprecipitates could only be detected after thermal annealing at a temperature around 500–600 °C, with substantial growth of the detected precipitates at higher temperatures. Hence, thermal annealing was performed at 800 °C with expectations of the formation of nano-oxide precipitates with a reasonable size. 

### 3.1. Different Morphologies of Synthesized Nanoprecipitates after Annealing at 800 °C

**Figure 1:**
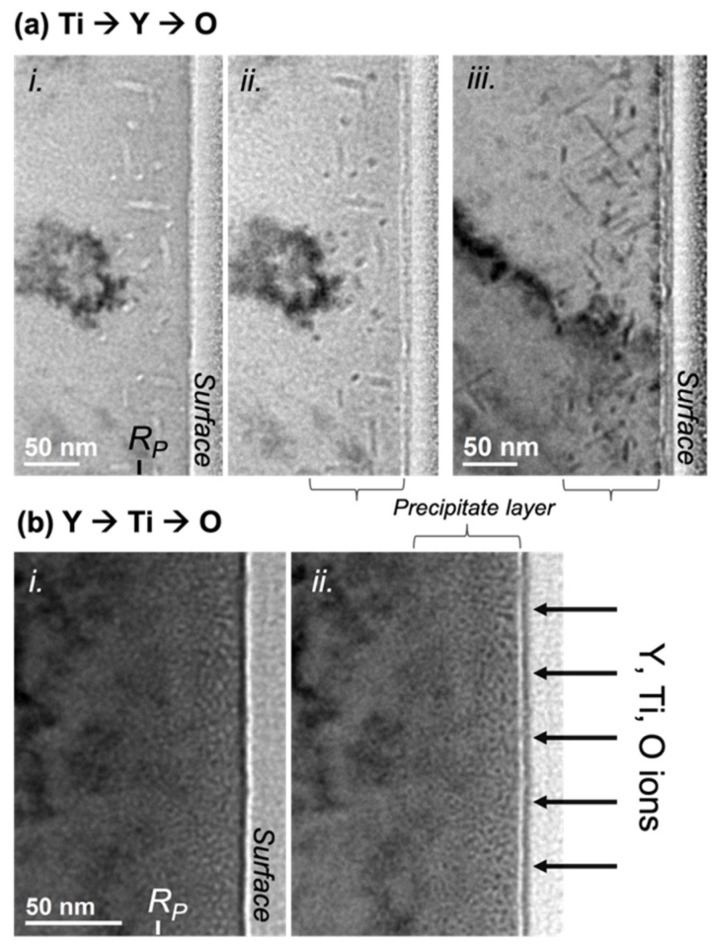
Cross-sectional defocused bright field micrographs of Fe-10%Cr samples implanted with Ti, Y, and O ions at room temperature and subsequently annealed at 800 °C for 2 h. Ion implantations were performed in the sequential orders (**a**) Ti ➔ Y ➔ O and (**b**) Y ➔ Ti ➔ O. Precipitates are observed in regions indicated with curly braces (“precipitate layer”), close to the mean projected range of implanted ions, R_p_. Precipitates are evidenced by using Fresnel contrast. In picture (i), the precipitates are observed in underfocus conditions, where they appear light with a contrasting surrounding Fresnel fringe. They are otherwise evidenced in overfocus conditions with reverse contrast in pictures (ii,iii). Pictures (i,ii) are from the same area, whilst picture (iii) shows a different grain of the thin foil. Irregular dark contrasts, visible in every picture, are induced by deformation of the thin foil.

Figure 1 displays the comparative results of a thermal annealing performed at 800 °C after the sequential ion implantations Y ➔ Ti ➔ O and Ti ➔ Y ➔ O. Nanoprecipitates are evidenced in both cases, but with markedly differing shapes and sizes. When Ti is implanted first, as shown in Figure 1a, the precipitates are rod-shaped with a length of a few tens of nanometers, measured at up to 60–70 nm, and a section of a few nanometers in diameter, roughly between 3 and 8 nm. This nanoprecipitate morphology is reminiscent of the Cr_2_O_3_-elongated precipitates obtained in the case of Ti and O implantation [25]. In every studied grain, there appears to be at least three preferential orientations for the long dimension of the precipitates. In Figure 1a(i,ii), showing the same area with different defocus, the precipitates are seemingly observed parallel and, possibly, perpendicular to their section. Figure 1a(iii) shows another grain of the specimen with a different set of precipitate orientations as compared to the implanted surface. 

In contrast, when Y is implanted first, as in Figure 1b, the precipitates appear roughly spherical with a diameter of about 2 to 3 nm. In addition, although no accurate density measurements were performed, the density of nanoprecipitates in this latter case appears significantly higher than in the case of Ti ➔ Y ➔ O implantation, assuming that both FIB thin foils have fairly similar thicknesses. In both cases, the nanoprecipitates are located in a layer of material that roughly matches the expected ion range: They are observed up to 80–90 nm for the Ti ➔ Y ➔ O case and up to 60 nm, with features more pronounced within the first 40 nm, for the Y ➔ Ti ➔ O case.

### 3.2. Case 1: Ti ➔ Y ➔ O Sequential Implantation

#### 3.2.1. Compositional Analysis of the Ti ➔ Y ➔ O Nanoprecipitates after Annealing at 800 °C

**Figure 2:**
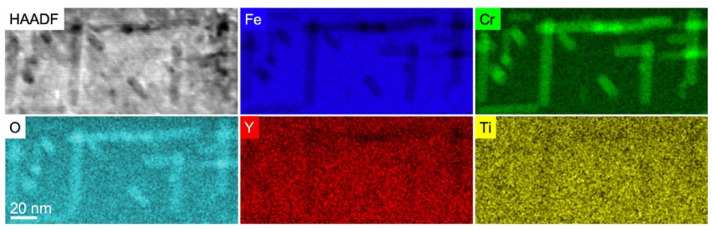
STEM-HAADF and STEM-EDX imaging of FeCr implanted with Ti, Y, and O ions in the sequential order Ti ➔ Y ➔ O and subsequently annealed at 800 °C for 2 h. The oxide precipitates are observed with a dark contrast on a lighter background in the STEM-HAADF image.

Figure 2 shows elemental maps obtained by performing STEM-EDX on the cross-sectional FIB lamella extracted from the sample implanted in the sequential order Ti ➔ Y ➔ O and annealed at 800 °C for 2 h. These maps clearly show that the rod-shaped precipitates are enriched in Cr and O. Compared to the matrix, they present a marked depletion of Fe and a subtler depletion of the implanted metallic elements Ti and Y, in accordance with the fact that these metallic elements are present in a much lower concentration in the material. 

#### 3.2.2. Crystallographic Structure of Ti ➔ Y ➔ O Nanoprecipitates after Annealing at 800 °C

**Figure 3:**
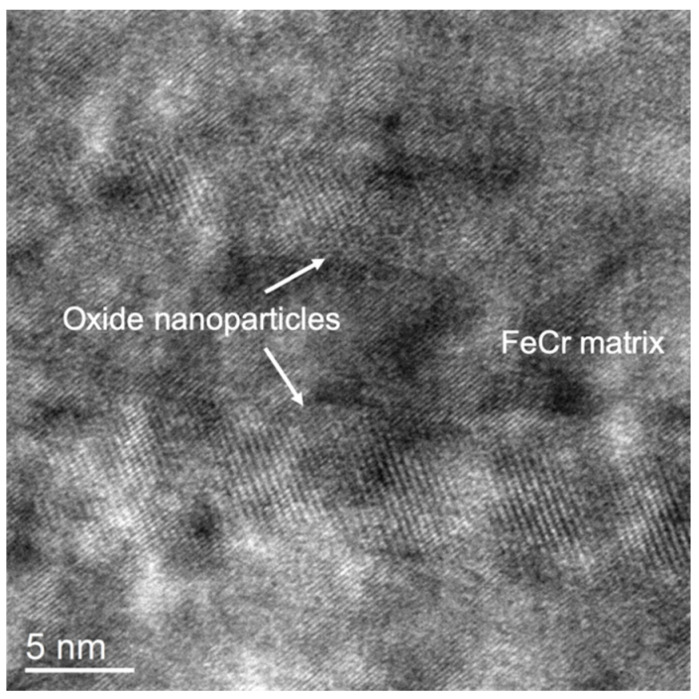
HRTEM picture of precipitates synthesized by Ti ➔ Y ➔ O implantations into FeCr followed by annealing at 800 °C for two hours.

Figure 3 shows an example of two precipitates, parallel to each other, imaged using HRTEM. These example precipitates show a pattern that contrasts with the surrounding matrix. When this pattern is clearly visible, the analysis of fast Fourier transforms (FFT) calculated on the precipitates and on the matrix usually enables the identification of the crystallographic structure and orientation of the precipitates and matrix. This analysis was performed on many HRTEM pictures in order to collect most of the crystallographic information in spite of difficulties inherent to the presence of a (Fe, Cr) spinel oxide on the surfaces of the thin foil [24], as well as the embedding of the nanoprecipitates within the FeCr matrix. 

The *bcc* structure of the FeCr matrix is first verified. The interplanar distances obtained from the matrix FFT are also in close agreement with theoretical values, showing that the TEM calibration is reasonably accurate, independently of the magnification used. In most pictures showing a clear pattern on the nanoprecipitates, however, the orientation of the matrix is often not fully identifiable, with, most of the time, only one visible family of planes with corresponding large spots in the FFT. An accurate calibration of the FFT distances using the matrix FFT as the reference was thus excluded in most cases. Consequently, for this specific specimen, the obtained values of interplanar distances are only approximate values, although very close to theoretical values, and are not displayed in the following. 

**Figure 4:**
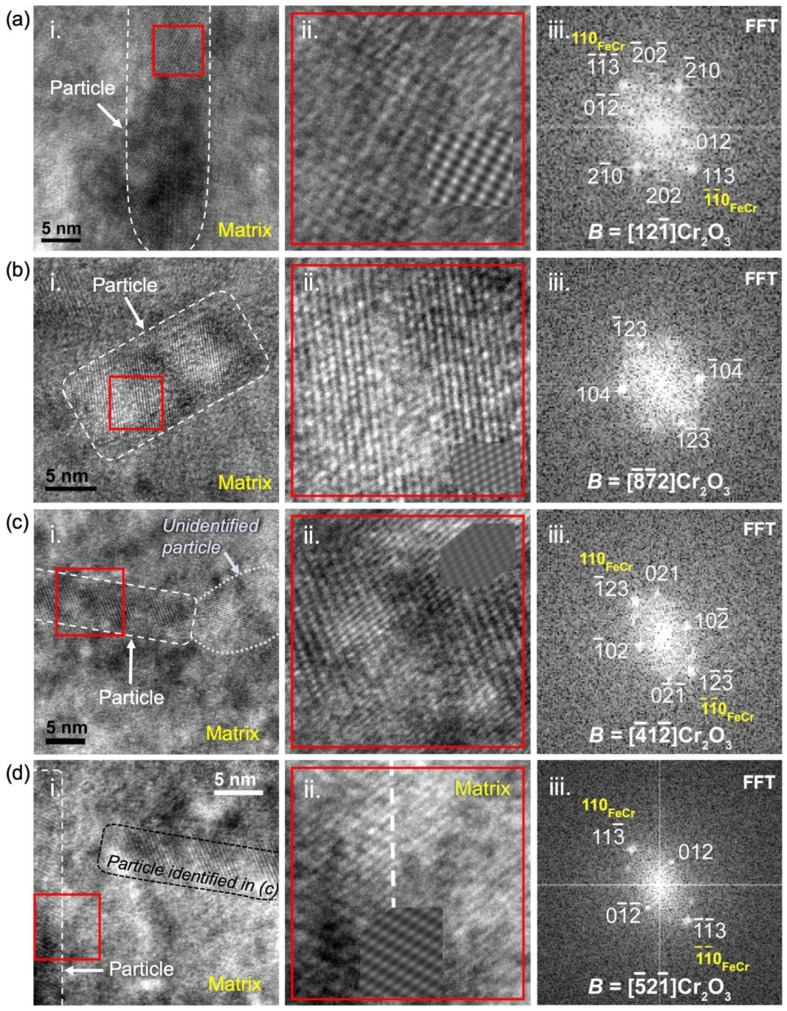
HRTEM imaging of precipitates synthesized with Ti ➔ Y ➔ O implantations and subsequent thermal annealing at 800 °C for two hours. A selection of four precipitates is displayed from sub-figure (**a**–**d**). In panel (i), the studied precipitates are outlined with a white dashed line. A red square indicates the region selected for FFT calculation, which is enlarged in panel (ii). The calculated FFT is reported in panel (iii) with identified structure and zone axis, as well as a corresponding indexation of spots deduced from the FFT analysis and simulation of the HRTEM pattern, which is reported as an insert in panel (ii). Unindexed spots on the FFT originate either from the matrix (unidentified reflections) or from a spinel surface oxide.

The results of the analysis of HRTEM pictures using FFT analysis and JEMS simulation are reported in Figure 4 for a number of nanoprecipitates. In Figure 4a, the measured interplanar distances, as well as the measured angles between the atomic planes from the FFT image, closely match those of the corundum hexagonal crystal structure of the Cr_2_O_3_ type viewed along the zone axis B = $\left[12\bar{1}\right]$. The FFT reported in panel (iii) perfectly matches the corresponding electron diffraction pattern calculated using the SingleCrystal software, with many visible families of planes. The HRTEM pattern is also well reproduced by the JEMS simulation, with a perfect orientation of planes and a correct ratio of distances, as shown by the insert in Figure 4a(ii). In addition, (113)Cr_2_O_3_ planes are found to be parallel to a family of (110)FeCr planes. The interplanar distance between these planes in ideal Cr_2_O_3_ and FeCr are 0.222 nm and 0.203 nm, respectively, which implies that every twelfth (110) plane of the matrix coincides with every eleventh (113) plane of Cr_2_O_3_. This discrepancy gives rise to relatively large spots on the FFT pattern. Finally, the precipitate in Figure 4a is elongated along a direction that appears parallel to $\left(\bar{1}23\right)$Cr_2_O_3_ planes.

The other precipitates reported in Figure 4(i), like most of the observed precipitates in this specimen, show FFT patterns that are also concordant with a crystallographic structure of the corundum hexagonal Cr_2_O_3_. The measured interplanar distances are very close to those expected for Cr_2_O_3_. In Figure 4b, the precipitate appears oriented along a zone axis $\left[\bar{8}\bar{7}2\right]$ with a HRTEM pattern well reproduced by the JEMS simulation. In this orientation, there is no obvious orientation relationship with the matrix, although some matrix spots could be identified in the FFT calculated in the surrounding of the precipitate (not shown). Like the precipitate in Figure 4a, the direction of elongation of the precipitate in Figure 4b is parallel to $\left(\bar{1}23\right)$Cr_2_O_3_ planes. In Figure 4c, the Cr_2_O_3_ precipitate appears oriented along a zone axis $\left[\bar{4}1\bar{2}\right]$. Again, the JEMS simulation, with a corresponding calculated HRTEM pattern reported in panel (ii), seems to confirm the structure and orientation. If this orientation is correct, the precipitate is elongated along $\left(12\bar{1}\right)$Cr_2_O_3_ planes. Moreover, the $\left(\bar{1}23\right)$Cr_2_O_3_ planes appear parallel to a family of (110) planes of the FeCr matrix. In Figure 4d, the characteristics of the FFT, calculated on the elongated vertical precipitate, are consistent with a precipitate with a corundum hexagonal Cr_2_O_3_ structure oriented along a zone axis $\left[\bar{5}2\bar{1}\right].$ The direction of elongation of the precipitate is not obvious but seems parallel to $\left(\bar{2}\;1\;12\right)$Cr_2_O_3_ planes. Similar to the precipitate of Figure 4a, the $\left(11\bar{3}\right)$Cr_2_O_3_ planes are found to be parallel to a family of (110)FeCr planes.

**Figure 5:**
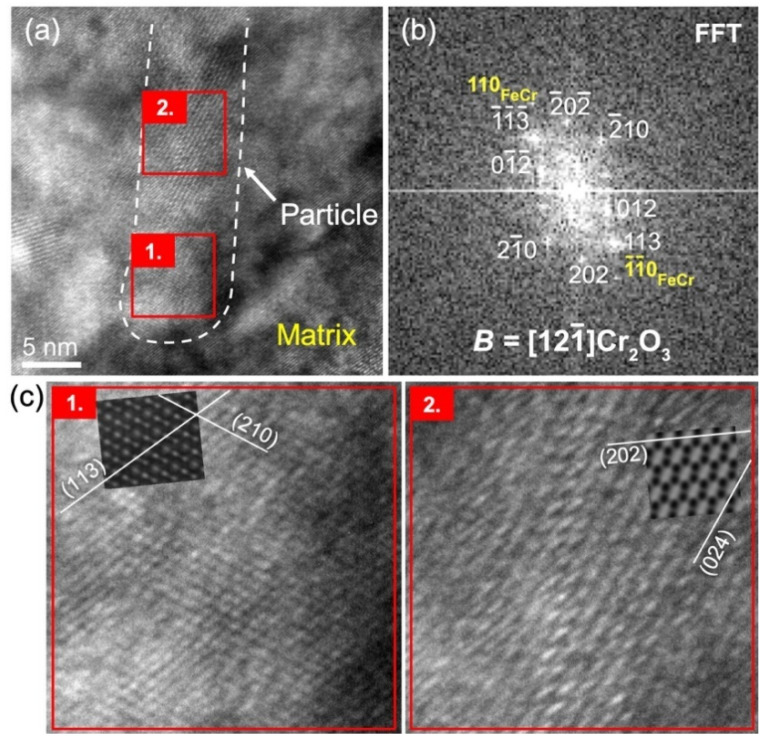
(**a**) HRTEM imaging of a precipitate with a corundum hexagonal Cr_2_O_3_ structure showing two different patterns in regions defined by red squares, labelled 1 and 2. The FFT calculated in regions 1 and 2 is identical and reported in (**b**), together with the identified direction of observation of the precipitate with corresponding spot indexing. Enlargements of both regions 1 and 2, as well as simulated HRTEM patterns, are provided in (**c**), with similar magnification for accurate comparison.

Another precipitate with an identified corundum hexagonal Cr_2_O_3_ structure is reported in Figure 5. Along this precipitate, as shown in Figure 5a,c, different HTREM patterns are visible, but the FFT stays unchanged and rather complex, with many visible spots. A perfect correspondence is found between this complex FFT and the calculated electron diffraction pattern in zone axis B = $\left[12\bar{1}\right]$. Moreover, both HRTEM patterns reported in Figure 5c are reasonably well reproduced by the simulation in this zone axis. This simulation requires a change in defocus value, as well as in thickness, to reproduce both patterns, which likely results from the fact that the precipitate is not parallel to the plane of the FIB lamella, as well as from local variations in the thickness of the FIB lamella. In the second pattern reported in Figure 5c, although the main pattern is correctly simulated with almost perfect orientations of planes and distances, the direction of stretching with respect to the large light spots is not well fitted. This stretching is likely induced by the superimposition with the matrix, which cannot be included in the simulation: It is indeed parallel to the visible (110)FeCr planes. During this study, this superimposition of many structures was found to frequently lead to the superimposition of different patterns and Moiré fringes, making the precipitate analysis particularly difficult. Figure 5c(2) additionally shows a slight distortion in the HRTEM pattern through the displayed picture, which is a fairly frequent occurrence in the observation of precipitates in this specimen. Hence, the simulated pattern can perfectly fit the HRTEM pattern on some parts of the precipitate and shows some slight discrepancies with the HRTEM pattern elsewhere. The edges of the precipitate in Figure 5 are difficult to accurately localize, but, as for the precipitate in Figure 4a with a similar direction of observation, this precipitate direction of elongation seems nearly parallel to $\left(\bar{1}23\right)$Cr_2_O_3_ planes. The same orientation relationship is also evidenced with (113)Cr_2_O_3_ planes parallel to a family of (110)FeCr planes.

Unfortunately, in the pictures where a clear HRTEM pattern is observed on the precipitates (thus enabling the identification of the precipitate structure and orientation), the matrix could not be fully characterized. Inversely, in pictures where the matrix is observed to be oriented along a determined zone axis, the precipitates could not be studied. In these latter pictures, the direction of elongation of the precipitates was observed to be parallel to $\left(031\right)$FeCr. More conveniently, the identification of the zone axes on pictures where the precipitates are seemingly observed parallel to their section, i.e., in the form of small disks as in the conventional TEM pictures of Figure 1a(i,ii), would resolve the direction of elongation of the rod-shaped precipitates. Diffraction patterns were unfortunately not acquired for such pictures. Apart from the precipitates with an evident corundum hexagonal Cr_2_O_3_ structure, some precipitates were also identified to have a spinel (Fe, Cr) oxide structure (not shown). This type of oxide has already been detected in the form of a thin layer on the surface of the implanted bulk specimens [24,25]. It is also present on the whole surfaces of the cross-sectional FIB lamellae but, in this case, usually distinguishable from the synthesized nanoprecipitates. The identified spinel precipitates are thus more likely precipitates close to the implanted surface, at the top of the implanted layer. Additionally, as underlined in Figure 4c(i), a few precipitates could not be satisfactorily described by either a corundum hexagonal Cr_2_O_3_ structure or a spinel (Fe, Cr) oxide structure. These precipitates are in the minority compared to the precipitates with corundum hexagonal Cr_2_O_3_ structures. Moreover, for some of these unidentified precipitates, a tolerance on the strain and distortion of the HRTEM pattern would probably suffice to relate them to either Cr_2_O_3_ or a spinel oxide.

### 3.3. Case 2: Y ➔ Ti ➔ O Sequential Implantation

#### 3.3.1. Compositional Analysis of Y ➔ Ti ➔ O Nanoprecipitates after Annealing at 800 °C

**Figure 6:**
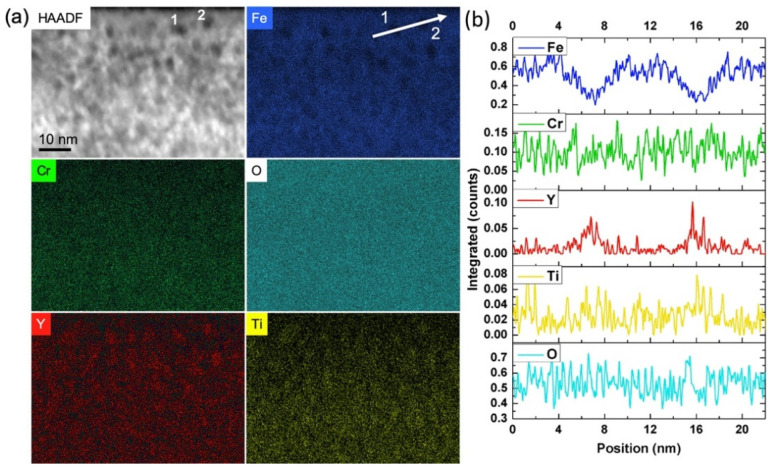
(**a**) STEM-HAADF and STEM-EDX imaging of FeCr implanted with Ti, Y, and O ions in the sequential order Y ➔ Ti ➔ O and subsequently annealed at 800 °C for 2 h. (**b**) One-dimensional line scans through two precipitates labelled 1 and 2 and highlighted in (**a**) on the Fe elemental map. The oxide precipitates are observed with a dark contrast on a lighter background in the STEM-HAADF image.

A compositional analysis was performed for precipitates obtained after implantation of Ti, Y, and O ions in the sequential order Y ➔ Ti ➔ O in a similar way as for the Ti ➔ Y ➔ O elongated precipitates (Section 3.2). The results of the STEM-EDX study performed after annealing at 800 °C are reported in Figure 6. The STEM-HAADF image, as well as the EDX maps, show what appears to be spherical or spheroidal precipitates of size that concord with conventional TEM observation, as reported in Section 3.1. As for the Ti ➔ Y ➔ O precipitates, a depletion of Fe is clearly seen at locations corresponding to nanoprecipitates, imaged as dark objects on a lighter background in the STEM-HAADF image. In contrast, the Cr and O maps appear homogeneous, whilst Y is evidently present in the precipitates, as highlighted by 1D line scans going through two precipitates and reported in Figure 6b. In a less obvious way, the Ti map also seems to show some Ti content inside the precipitates. Finally, the absence of Cr depletion within the precipitates suggests that they might contain a notable amount of Cr.

**Figure 7:**
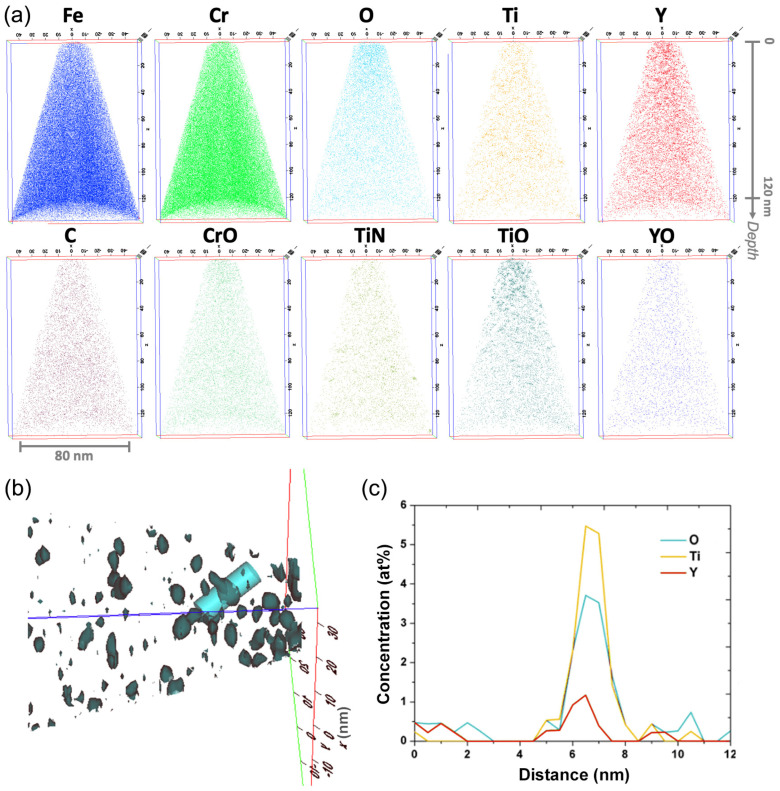
(**a**) Three-dimensional reconstructions of APT tips extracted from an FeCr specimen implanted with Y ➔ Ti ➔ O ions and annealed at 800 °C for two hours, showing the distributions of the evaporated Fe, Cr, O, Ti, Y, and C elements, as well as CrO, TiN, TiO, and YO molecular ions. (**b**) Three-dimensional image of TiO clusters represented using an iso-concentration surface of 0.8% TiO. (**c**) Concentration profiles along a cylinder running through a cluster, as represented in (**b**). The cylinder is 12 nm long.

This compositional analysis was complemented by APT experiments performed on three different APT tips extracted from the specimen implanted with Y ➔ Ti ➔ O ions and annealed at 800 °C, in particular to ascertain the presence of Y and Ti inside the precipitates. The 3D reconstructions of all three APT tips look similar; an example is displayed in Figure 7a. These reconstructions show a clear correspondence of inhomogeneities in Y, Ti, O, and TiO distributions in the implanted region, which is an indication of the formation of oxide clusters rich in Y and Ti. Concentration profiles through clusters obtained from TiO iso-concentration surfaces also clearly show the presence of Ti, Y, and O inside the precipitates. An example is represented in Figure 7b,c; similar results were obtained for different clusters located in the three studied APT tips. The absence of O detection inside the nanoprecipitates using EDX mapping thus appears to be surprising, but it can be explained by the presence of the spinel oxide on the whole surfaces of the FIB lamella, reducing the sensitivity to the O presence inside the embedded nanoprecipitates. 

Unfortunately, a proper identification of the crystallographic structure of the nanoprecipitates obtained with the sequential order of ion implantations Y ➔ Ti ➔ O could not be obtained due their very small size.

#### 3.3.2. Y ➔ Ti ➔ O Nanoprecipitates Resulting from Annealing at 1100 °C

**Figure 8:**
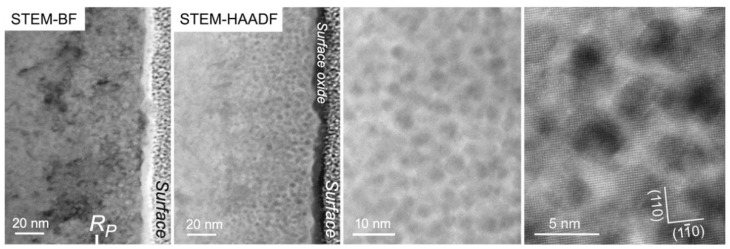
STEM-BF and STEM-HAADF imaging of an FeCr specimen implanted with Y ➔ Ti ➔ O ions and annealed at 1100 °C for two hours. The synthesized precipitates appear light on a darker background in the STEM-BF picture, and dark on a lighter background in STEM-HAADF pictures. The first two images are from the same area and are taken at the same magnification. The last picture taken at high magnification highlights the cuboidal shape of the precipitates.

To make the precipitates grow for clearer identification, a further annealing at 1100 °C for two hours was performed on bulk specimens implanted at room temperature in the sequential order Y ➔ Ti ➔ O. This temperature also corresponds to the temperature applied during the consolidation process in the conventional fabrication method of ODS steels. As shown in Figure 8, when observed at relatively low magnification, the resulting precipitate shape looks similar to the shape of precipitates observed after annealing at 800 °C, i.e., more or less spherical. The precipitates are actually facetted with an almost cuboidal shape, as shown via imaging at high magnification. They are slightly larger after annealing at 800 °C, with a size ranging roughly between 2 and 5 nm, and are observed to be up to 80 nm in depth, with a rough decreasing gradient of the precipitate size up to this depth.

**Figure 9:**
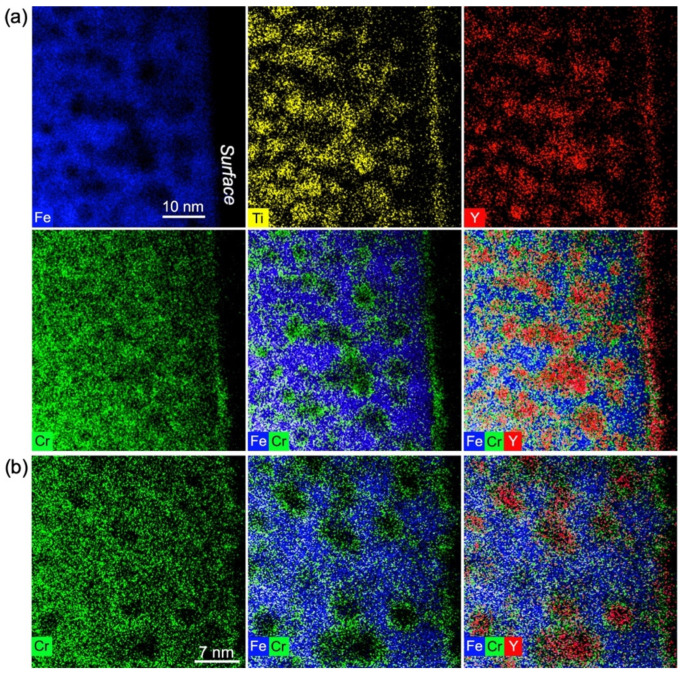
STEM-EDX imaging of precipitates synthesized by Y ➔ Ti ➔ O ion implantation into FeCr, subsequently annealed at 1100 °C for two hours. Two different areas are represented with different magnifications in (**a**,**b**).

Figure 9 shows the results of STEM-EDX analysis after annealing at 1100 °C. As observed after annealing at 800 °C, the dark, rounded patches observed in STEM-HAADF correspond to clear localized enrichments in Y and Ti. On the O map (not shown), O-rich patches are also observed to coincide with the rounded features observed in the Y and Ti maps, but with larger sizes. However, as explained earlier, the sensitivity of O detection inside the precipitates is relatively poor in these experiments. Unlike previous observations after annealing at 800 °C, the Cr map looks inhomogeneous, with ring-like patterns indicative of a Cr shell around the precipitates; this is more obvious in the superimposition of Fe and Cr maps, where Cr enrichments in the form of circles are mostly observed in regions depleted of Fe. The Cr shell can be better observed between two adjacent precipitates, where Fe is not observed and where only the presence of Cr is detected. The areas depleted of both Fe and Cr inside the Cr circles coincide with Y and Ti-rich regions. 

**Figure 10:**
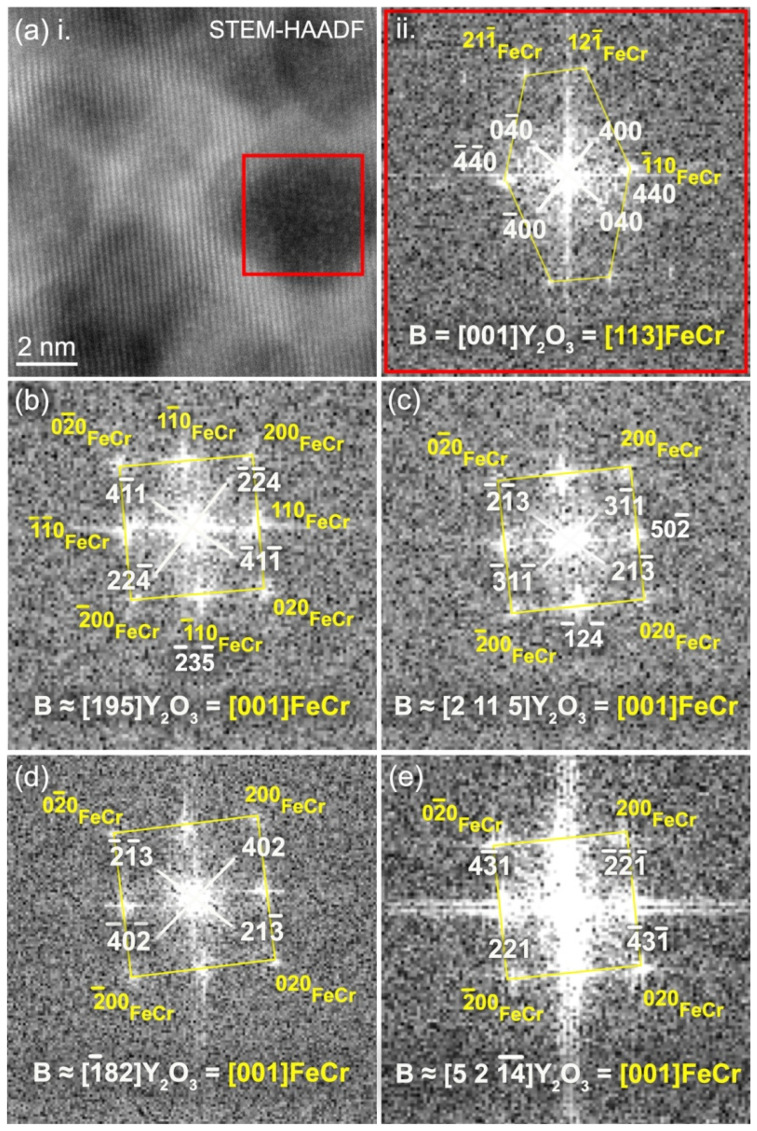
(**a**) In panel (i): STEM-HAADF picture of FeCr implanted with Y ➔ Ti ➔ O ions and annealed at 1100 °C for two hours showing a few precipitates embedded in the FeCr matrix. The FFT calculated on a precipitate highlighted in this panel is reported in panel (ii), along with results of the crystallographic analysis. (**b**–**e**) FFT calculated on other precipitates selected in one grain of the matrix that was exactly oriented along zone axis [001].

STEM-HAADF performed on the Y ➔ Ti ➔ O specimen unfortunately does not enable a proper visualization of the atomic column arrangements inside the embedded nanoprecipitates, partly because of a very high density of produced nanoprecipitates, as evidenced in Figure 8. However, by sweeping a live FFT selection square through the obtained HAADF pictures, some FFT spots are observed to only appear when the selection square encompasses rounded precipitate features; they can thus undoubtedly be attributed to the precipitates. Some examples of FFT containing some precipitate spots are reported in Figure 10. In these examples, the matrix is obviously oriented along easily identifiable zone axes, i.e., B = [113] in Figure 10a and B = [001] in Figure 10b–e. This enables an accurate calibration for the measurement of interplanar distances in the FFT patterns. The first FFT pattern reported in Figure 10a(ii), was calculated on the precipitate highlighted in the STEM-HAADF picture in Figure 10a(i), and shows two pairs of precipitate spots that are 90° apart and that correspond to equal interplanar distances measured at 0.280 nm. This pattern is specific to cubic structures and completely excludes precipitates with corundum hexagonal Cr_2_O_3_ structures, which is also supported by compositional analysis, as well as the morphology of the precipitates: The Cr_2_O_3_ precipitates indeed seem to always occur with elongated rod-like shapes (see Section 3.2 of the present paper [25]). This also excludes a likely (Y,Ti) oxide, Y_2_TiO_5_, which crystallizes into a complex orthorhombic structure that is stable up to 1330 °C [33]. The interplanar distance *d* = 0.280 nm, however, does not enable us to distinguish between either precipitates of the cubic Y_2_O_3_ structure (or similar structures, including cubic Y_2_Ti_2_O_7_) oriented in the zone axis B = [001] (i.e., (040)Y_2_O_3_ planes with a theoretical value of distance, *d*_040_ = 0.267 nm) or precipitates with a spinel structure, also in zone axis B = [001] (i.e., (220) planes with a theoretical value *d*_220_ = 0.301 nm for Fe_3_O_4_). The second FFT pattern displayed in Figure 10b shows two pairs of precipitate spots that are 86° apart and correspond to values of interplanar distances *d* = 0.217 nm and *d* = 0.251 nm. In this case, the spinel structure is excluded since it cannot reproduce this FFT pattern with a correct angle or d-spacings. It is, however, perfectly compatible with precipitates with a Y_2_O_3_ structure oriented in zone axis B = [195], with (411) and (224) planes theoretically separated by 85° and with theoretical interplanar distances *d*_411_ = 0.252 nm and *d*_224_ = 0.218 nm. The third reported FFT pattern in Figure 10c also confirms the cubic Y_2_O_3_ structure: the measured distances and angle, *d* = 0.319 nm, *d* = 0.285 nm, and 83°, perfectly fit zone axis B = [2 11 5]Y_2_O_3_, where $\left(3\bar{2}2\right)$ planes ($d_{3\bar{2}2}$ = 0.322 nm) and $\left(21\bar{3}\right)$ planes ($d_{21\bar{3}}$ = 0.286 nm) should be observed 81° apart. This, in particular, firmly excludes spinel oxide precipitates since the interplanar distance *d* = 0.319 nm does not exist in the spinel. Two other examples are provided in Figure 10d,e. In Figure 10d, the FFT pattern cannot be reproduced by either the Cr_2_O_3_ or the spinel structures. D-spacings of 0.242 nm and 0.284 nm are found with an angle of 84°, which is related to the Y_2_O_3_ structure observed close to the zone axis $\left[\bar{1}82\right]$ (theoretical interplanar distances *d*_402_ = 0.239 nm and $d_{21\bar{3}}$ = 0.286 nm, with planes 84° apart). In Figure 10e, d-spacings of 0.209 nm and 0.358 nm are observed with an angle of 80°; this closely matches the theoretical interplanar distances of planes (221) and $\left(4\bar{3}1\right)$ (*d*_221_ = 0.357 nm and $d_{4\bar{3}1}$ = 0.210 nm), which should theoretically be observed 80° apart when Y_2_O_3_ is oriented close to the zone axis $\left[5\;2\;\bar{14}\right]$.

This FFT analysis does not enable us to differentiate between Y_2_O_3_ precipitates and pyrochlore Y_2_Ti_2_O_7_ precipitates that crystallize with similar cubic structures. Nevertheless, we can highlight that all the measured interplanar distances related to precipitate spots almost perfectly match the theoretical values of the cubic Y_2_O_3_ structure, and that the occurrence of pyrochlore precipitates should substantially reduce these values (the lattice parameters of Y_2_O_3_ and Y_2_Ti_2_O_7_ are *a* = 0.10704 nm and *a* = 0.10188 nm, respectively). The FFT calibration performed using matrix distances thus indicates that the precipitates are more likely cubic yttria (Y_2_O_3_) precipitates with a certain amount of Ti, as shown by the STEM-EDX results, rather than stoichiometric pyrochlore precipitates. The absence of other elements, such as Cr, inside the precipitates cannot be firmly ascertained, but they should not be present in significant concentration, as suggested by the STEM-EDX experiments (see Figure 9). In particular, the Cr depletion visible in the core of the precipitates in Figure 9 suggests that the precipitates are Y_2_O_3_ precipitates with a Cr shell; they will be accordingly referred to as Y_2_O_3_ precipitates in the following. The presence of other (Y, Ti) oxides in the Fe-10%Cr matrix is not completely excluded either, since all the precipitates could not be studied; however, it should, at best, be marginal compared to the high occurrence of precipitates with a Y_2_O_3_ structure. This analysis also shows that the yttria precipitates can take many different orientations in one grain of the matrix, here shown oriented along the zone axis [001], which possibly enhances the difficulty in observation and analysis of those precipitates that appear in high density and are rarely isolated in TEM pictures. Moreover, the different directions of observation of the precipitates reported in the Figure 10 are apparently unrelated, although the directions [195] and [2 11 5] in Figure 10b,c are separated by only 6°.

Some orientation relationships between the precipitates and the matrix could also be evidenced. In Figure 10a, with [001]Y2O3 || [113]FeCr (the symbol “||” meaning that both directions are parallel to each other), the (440) planes of the precipitate are observed to be parallel to $\left(\bar{1}10\right)$ matrix planes. In this orientation, the precipitate facets are observed to be parallel to {400}Y_2_O_3_ planes (see the STEM-HAADF image in Figure 10a), but no relation to matrix planes can be inferred. In Figure 10b, the relation $\left(2\bar{3}5\right)$Y_2_O_3_ || $\left(1\bar{1}0\right)$FeCr is observed. In Figure 10c, the relations $\left(\bar{1}2\bar{4}\right)$Y_2_O_3_ || $\left(1\bar{1}0\right)$FeCr and $\left(50\bar{2}\right)$Y_2_O_3_ || (110)FeCr can be deduced, the first with a significant mismatch visible on the FFT, and the second with perfectly matching spots. In Figure 10d, although corresponding spots are not clearly seen, it appears that the $\left(61\bar{1}\right)$Y_2_O_3_ planes should be parallel to the matrix (110) planes with a relatively strong mismatch. With the matrix orientation B = [001], the precipitate facets are clearly observed to be parallel to matrix {200} planes (see picture on the right of Figure 8). A relation between these facets and the precipitate planes is less obvious, although they appear to be parallel to {400}Y_2_O_3_ planes in Figure 10a, which would then imply {200}FeCr || {400}Y_2_O_3_. In the literature, a cuboidal shape of Y_2_O_3_ precipitates was observed in Fe-Cr-Ti-Y_2_O_3_ after annealing at 1300 °C for 1 h and was ascribed to the combined elastic anisotropy of the cubic matrix and the partial coherency of interfaces [34]. In that study, as in the present case, although the orientation relationships between the matrix and precipitates seem to differ, with apparently looser conditions with respect to the precipitate planes and directions in our case (i.e., no general orientation relationship), the main facets of the Y_2_O_3_ precipitates appear to be parallel to the same matrix planes, i.e., {200}FeCr planes. A similar observation was furthermore made in an Fe-Al-based ODS alloy with Y_2_O_3_ precipitate facets parallel to {100}, {110}, and {112} matrix planes. This faceting was observed to occur when there was a near coincidence of Y_2_O_3_ planes with low index FeAl planes, suggesting that the faceting mechanism is probably a diffusional process enhanced by the formation of low energy interfaces rather than a reaction of the yttrium oxide with the matrix during the mechanical alloying process [35].

In our specific case, the FeCr matrix appears to “constrain” the precipitation, forming interfaces with the Y_2_O_3_ precipitates along its {200} planes, and, most importantly, with its {110} planes, i.e., the planes of highest atomic density, almost always observed to be parallel to precipitate planes (this last observation is also true for Cr_2_O_3_ precipitates observed after Ti ➔ Y ➔ O (see Section 3.2.2) as well as Ti ➔ O implantation experiments [25]). The precipitates, on the other hand, seem to have many possibilities of orientation, with many possible yttria planes parallel to matrix {110} planes.

**Figure 11:**
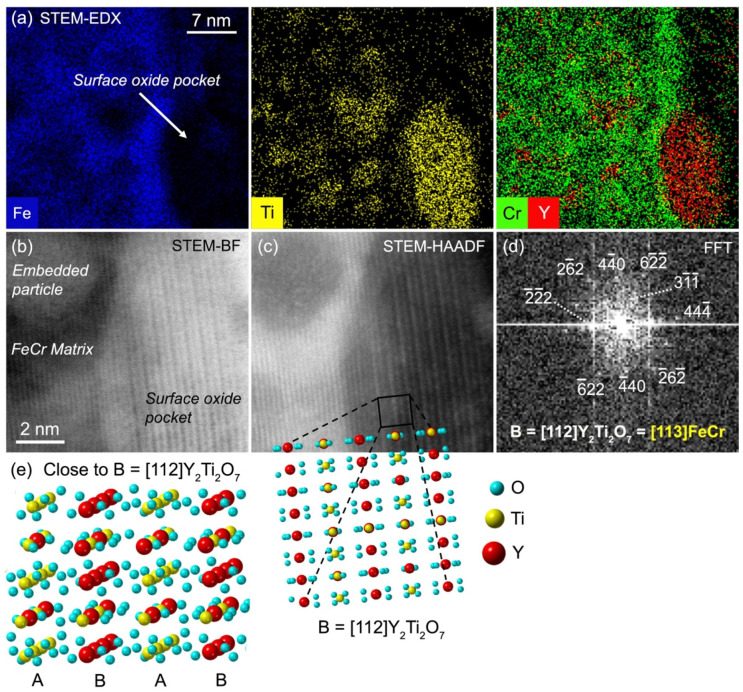
Analysis of an oxide pocket on the surface of an Y ➔ Ti ➔ O-implanted FeCr specimen after annealing at 1100 °C: (**a**) compositional analysis with STEM-EDX; (**b**) STEM-BF image; (**c**) STEM-HAADF image; (**d**) FFT calculated on the surface oxide pocket from the STEM-HAADF image, along with results of crystallographic analysis; (**e**) periodic arrangements of atoms observed in Y_2_Ti_2_O_7_ oriented along the zone axis B = [112]. Alternations of different (222) planes, labelled A and B, are shown.

In addition to these embedded precipitates, a surface oxide pocket showing similar features in STEM-EDX maps, i.e., clear Y and Ti enrichments as well as Fe depletion, was analysed. The surface oxide pocket is relatively large compared to the embedded nanoprecipitates: it is roughly 17 nm long and 7 nm wide. The STEM pictures of this surface pocket, as well as results of the analysis, are reported in Figure 11. The distances in the FFT were accurately measured from calibration on the surrounding FeCr matrix oriented along zone axis [113] (not shown). The calculated FFT reported in Figure 11d shows a clear rectangular pattern with measured interplanar distances of 0.154 nm, 0.299 nm, and 0.176 nm. Moreover, an angle of 59° is measured between the longest and shortest of these distances. This pattern, as well as the STEM pictures, are best described by the cubic structure of either Y_2_O_3_ or pyrochlore Y_2_Ti_2_O_7_ precipitates oriented along zone axis [122]. In this zone axis, the 59° angle is theoretically expected between $\left(22\bar{2}\right)$ and $\left(6\bar{2}\bar{2}\right)$ planes. Unlike the observations made for the embedded precipitates, the measured d-spacings are clearly in favour of pyrochlore precipitates: they closely match the theoretical values $d_{6\bar{2}\bar{2}}\;$= 0.154 nm, $d_{22\bar{2}}\;$= 0.294 nm, and $d_{4\bar{4\;}0}$= 0.180 nm in Y_2_Ti_2_O_7_ and are significantly smaller than those in Y_2_O_3_. This is also supported by an obvious alternation of parallel $\left(22\bar{2}\right)$ planes with contrasting intensities in the STEM-HAADF picture in Figure 11c. This reveals an alternation of cationic planes with different average atomic numbers. This alternation of contrasting planes cannot be explained by a close-to-pure Y_2_O_3_ structure and composition of the precipitates, where $\left(22\bar{2}\right)$ planes should be equivalent in density, average atomic number, and positioning of atoms. This, however, is in perfect agreement with the alternation of $\left(22\bar{2}\right)$ cationic planes A and B observed in the pyrochlore structure, with A = Ti  Y + Ti  Ti  Y + Ti … and B = Y  Y + Ti  Y  Y + Ti …, as shown in Figure 11e. The STEM-HAADF imaging additionally shows that the (222) planes of the pyrochlore pocket are parallel to (110) planes of the FeCr matrix, and the pyrochlore pocket also appears elongated along these matrix planes. Another pyrochlore surface pocket observed with the FeCr matrix oriented along the zone axis [001] shows its (004) planes parallel to matrix (110) planes (not shown). In the Figure 11, the interface between the pyrochlore pocket and the matrix is obviously enriched in Cr in a similar way to the shells surrounding the embedded precipitates. This interface appears to follow the *bcc* structure of the matrix. Its relatively dark shade when observed in STEM-HAADF may indicate the presence of another lighter element in addition to Cr and Fe, possibly O, which, unfortunately, cannot be confirmed with STEM-EDX experiments in the present conditions. A loss of HAADF intensity might additionally result from disorder through a reduction of electron channelling [36]. 

Pyrochlore Y-Ti oxide and cubic yttria having close structures, and with the atomic density of pyrochlore being higher (~8.3 × 10^22^ at/cm^3^ compared to 6.5 × 10^22^ at/cm^3^) and very close to that of the FeCr matrix (~8.5 × 10^22^ at/cm^3^), there is, a priori, no geometrical reason for the presence of the pyrochlore structure on the surface, whilst yttria precipitates enriched in Ti are embedded in the matrix. This is thus the likely result of the availability of Ti in the surroundings, with probably much more Ti close to the surface. SIMS experiments on Ti-implanted FeCr specimens have indeed shown that thermal annealing at 1100 °C induces a strong shift of the Ti concentration profile towards the surface, with the peak Ti concentration located at the surface after a two-hour annealing [32]. A similar effect is observed in the Y concentration, although to a lesser extent [32]. Thus, the highest concentrations of Y and Ti, as well as the highest Ti/Y ratio, should probably be on the surface. This can also explain the observed decreasing gradient in the size of the precipitates, with larger precipitates observed close to the surface. 

## 4. Discussion

Conventional ODS steel fabrication is performed by simultaneously mixing alloying elements. In the present ion beam experiments, implantations of Y, Ti, and O ions were performed sequentially into the FeCr matrix. The successive ion implantations led to the formation of oxide nanoprecipitates only detectable after thermal annealing. The results displayed in the previous sections and summarized in Table 1 show that the sequential order of ion implantations is decisive in the determination of the specific oxide that precipitates after thermal annealing. The high sensitivity of the final microstructure to the order of metal ion implantation, with similar elemental composition of the implanted matrix at the final step of ion implantation, indicates that the atomic-scale processes determining the subsequent precipitate nucleation pathways operate already during ion implantation at RT.

When Ti is implanted first, elongated, rod-shaped precipitates with a corundum structure are identified. It is almost impossible to differentiate between Cr_2_O_3_ and Fe_2_O_3_ precipitates with the crystallographic analysis because the lattice parameters of these oxides of similar structure are extremely close [37]. However, the qualitative compositional analysis shows a strong enrichment in Cr, which allows us to assume that the precipitates are mostly Cr_2_O_3_ precipitates, although some Fe content cannot be excluded. These results are identical to the results obtained after implantation of Ti and O ions into FeCr [25], except that, in the present case of triple-ion implantation, the precipitates appear free of Ti after annealing at 800 °C, whilst Ti was observed to enrich the Cr_2_O_3_ precipitates after a similar annealing. In the literature, Cr_2_O_3_ is often observed to form in FeCr or FeNiCr matrices as a result of weeks-long experiments of corrosion in air or water. It has been, for example, observed in ODS matrices [38], austenitic stainless steels [39], and nickel-based alloys (Inconel600, for example [40]). The occurrence of Cr_2_O_3_ seems particularly important in ODS steels, as compared to other ferritic/martensitic steels, where the presence of Y-Ti-O precipitates has been suggested to reduce the amount of chromium needed to form Cr_2_O_3_ by serving as nucleation sites for the oxide [41], though the detailed mechanisms of Cr_2_O_3_ formation have not been discussed. 

The similarity in the outcome of the present experiment with Ti ➔ Y ➔ O ion implantation, followed by high-temperature annealing, and the earlier experiment without the intervening Y beam [25], indicates the similarity of the involved microstructural mechanisms of oxide precipitation. As suggested in Ref. [25], based on the first-principles modelling results, the nucleation of corundum Cr_2_O_3_ is most probably related to the formation of chromium local precipitation zones (LPZ), which serve as precursors for internal oxide nuclei formation at the subsequent O ion implantation stage. In order to serve as Cr_2_O_3_ nucleation sites, these zones should contain a sufficient number of chromium atoms (at least eight), which is hard to achieve unless the Cr concentration in the matrix is sufficiently high (the known data on Cr precipitation and oxidation in ferritic-martensitic steels suggest the lower threshold of Cr concentration at the level of ~15 at.% [25]). The reason is that Cr atoms in iron matrices tend to repel each other at separations up to the third-nearest neighbour [42]. While the involved extra energies required to put Cr atoms close together are relatively low (~0.1–0.25 eV), they are significant enough to prevent Cr clustering at RT in insufficiently concentrated Fe/Cr alloys.

The existence of a Cr concentration threshold for efficient chromium LPZ formation and subsequent oxide nucleation implies that the pristine concentration of 10 at.%Cr in our samples is insufficient to promote the formation of chromium oxide nuclei. However, upon the addition of extra 5 at.% of titanium, which behaves in the iron matrix very similarly to chromium in terms of solute–solute and solute–oxygen interaction [42], the total Cr + Ti concentration increases up to ~15% (or even higher due to Ti redistribution towards the sample surface), reaching the required threshold. As can be judged from the results of the experiment with Ti ➔ O implantation [25], the necessary reordering of the minor alloy components, which creates a sufficiently large number of nucleation sites for Cr_2_O_3_ precipitates that are able to grow later on at the high-temperature annealing stages and be detected by TEM, is over already by the end of Ti ion implantation and prior to oxygen implantation. The results of our present experiment demonstrate that an additional Y ion implantation between Ti and O does not affect much LPZs formed at the stage of Ti implantation. The explanation is rather evident. While titanium and chromium performance in the iron matrix is quite similar in terms of their interaction and mobility, Y atoms implanted in the iron-based matrix behave very differently. Yttrium is a strongly oversized solute in Fe/Cr alloys, so that, in irradiation environments, Y atoms tend to trap vacancies, making strongly bound Y-V complexes with Y atoms located in between two empty lattice sites [17,42]. Such complexes are essentially immobile via either interstitial or vacancy mechanisms at RT and thus do not participate in the alloy component reordering. That is, in contrast to titanium, Y implantation does not promote local Cr enrichment in LPZs, but at the same time, it does not break LPZs already formed at the preceding Ti implantation stage unless an Y atom stops exactly inside an LPZ (which can hardly spoil all LPZs for the implanted Y concentration of 6 at.% in the peak). In fact, the only effect of Y implantation in this case is expected to be Ti immobilization.

**Figure 12:**
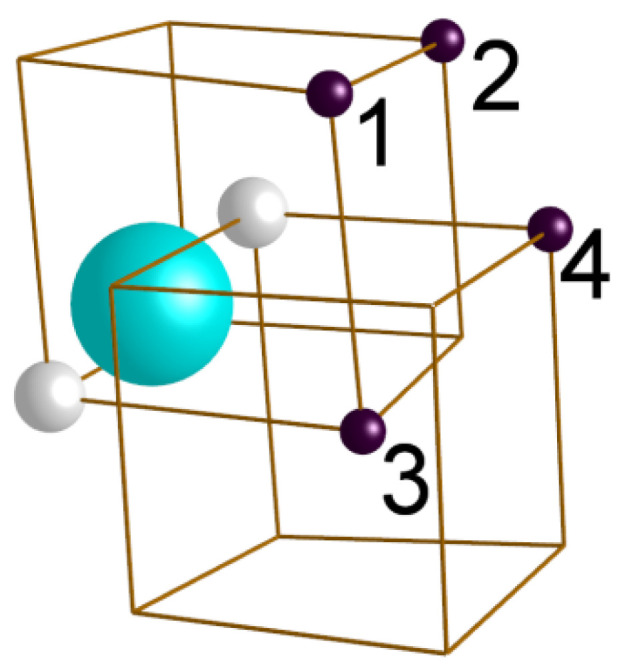
Symmetrically non-equivalent positions of a Ti atom with respect to a Y-V pair in *bcc* iron. The Y atom is marked with a blue sphere, and empty sites are indicated with smaller white spheres. The brown spheres represent Ti atoms located in positions numbered from 1 to 4, as referenced in Table 2.

Indeed, even though the earlier studies on solute–solute interactions in iron [18,42] suggested that the interaction between substitutional Y and Ti atoms is weakly repulsive, with binding energies of ~−0.3 eV and ~−0.15–0.2 eV at the first and the second-nearest neighbour separations, respectively (here, the minus sign implies that the energy of two isolated atoms is lower than that of a close solute pair), they considered Y to be an onsite atom. For a more realistic Y-V complex, DFT-based calculations predict that its interaction with a nearby Ti atom is attractive; see Table 2 and Figure 12 (calculation details are summarized in the table header). Even if quite modest, at room temperature, the temperature at which the ion implantation was performed, this binding is strong enough to suppress Ti-Y pair dissociation. Having in mind a relatively high mobility for Ti atoms during Y implantation due to radiation-enhanced diffusion via interstitial-mediated mass transport, and the fact that the distance between Y atoms in the implanted layer by the end of implantation is only a few nanometres, it is quite reasonable to expect that all Ti atoms that do not escape the Y-implanted layer will become trapped on Y atoms, making small Y-Ti clusters. 

At the final O ion implantation stage, both LPZs and Y-Ti complexes are expected to trap interstitial O—which is quite mobile at RT with its estimated migration energy of ~0.5–0.6 eV [20,42]—and transform into oxide cluster nuclei. The binding energies of O in such clusters are quite high (e.g., ~0.75 eV for O-Y bonding [42] and >1.5 eV for O atoms trapped in a Cr_2_O_3_ nucleus [25]) and, thus, the oxide precursors are expected to survive heating to high temperatures. It is quite evident, however, that the growth of that or another cluster type will be limited by the mobility of metal atoms that constitute the oxide. Chromium mobility in high-temperature annealing conditions is limited by vacancy transport and thus starts at roughly 600 °C [25,32], where thermal vacancy mediated self-diffusion usually becomes noticeable in ferritic steels. Y-Ti-based oxide clusters at such temperatures have no chance to grow because Y remains practically immobile in iron up to at least 800 °C [32]. Thus, it seems natural that intermediate Y implantation does not visibly influence the oxide precipitation pattern observed without Y implantation [25]. 

The only noticeable difference in the present experiment, as compared to Ref. [25] (Ti ➔ O implantation) is that precipitates obtained after annealing at 800 °C in the Ti ➔ Y ➔ O specimen are free of Ti, whereas, without yttrium addition, the oxide particles contain a detectable Ti contribution. The trapping of Ti atoms in Cr_2_O_3_ with the formation of mixed (Cr,Ti) oxide should be energetically favourable because the enthalpy of formation of corundum Cr_2_O_3_ is higher than that of Ti_2_O_3_ [43]. Accordingly, the oxide enrichment in Ti took place in the Ti ➔ O experiments as Ti became mobile at an annealing temperature close to 800 °C [24,25]. The lack of Ti in Cr_2_O_3_ in the present study provides a strong hint that Ti at this temperature remains efficiently trapped in invisible (Y,Ti) oxide clusters.

The change in metal implantation order changes the oxide formation pattern drastically. When Y is implanted first, nanometric spheroidal or cuboidal precipitates are observed to be embedded in the *bcc* FeCr matrix. Chromium oxide formation is totally suppressed, and Y-based oxide precipitates become visible after annealing at 800 °C. The origin of this effect is evident. As already discussed, the implanted yttrium is immobile and does not promote the formation of chromium LPZs, while Ti implantation after yttrium into the same region where Y is located results in quick Ti trapping on Y atoms and immobilization. As a result, the efficient Cr concentration is not augmented by equally mobile titanium contributing to LPZ formation but rather gradually decreased due to the implantation of additional 10 at.% of Y and Ti. The lack of proper places for chromium oxide nucleation is subsequently manifested in the lack of visible Cr_2_O_3_ precipitates after high-temperature annealing. 

Post-annealing observations point towards a cubic Y_2_O_3_ structure for the precipitates with a certain amount of Ti, whilst stoichiometric pyrochlore Y_2_Ti_2_O_7_ and Y_2_TiO_5_ precipitates seem to be missing inside the bulk of the specimen. The precipitates are also observed to be surrounded by a Cr shell. This kind of Cr-enriched shell was recurrently reported as a characteristic of (Y,O)-rich nanoclusters in ODS steels [10,15,44,45]. In addition to the embedded precipitates of Y_2_O_3_ structure, an oxide pocket on the surface of the implanted Y ➔ Ti ➔ O specimen clearly shows a Y_2_Ti_2_O_7_ pyrochlore structure, which probably highlights the importance of the surrounding Ti amount in the formation of pyrochlore since the Ti concentration is expected to peak at the surface after annealing at 1100 °C. If such is the case, an implantation of Ti at higher fluence would likely lead towards the formation of stoichiometric pyrochlore precipitates that have similar structure to and higher atomic density than yttria precipitates, as frequently observed in Ti-doped ODS steels [34,46,47,48,49]. In these steels, the main oxide precipitates have been demonstrated to be Y_2_Ti_2_O_7_ and Y_2_TiO_5_ [9,46,50,51], whilst the shortage of Ti has been shown in a number of instances to lead to the formation of Y_2_TiO_5_ [46,52]. Thus, with both Ti and Y implanted in the FeCr matrix, Y_2_TiO_5_ precipitates would be expected, rather than Y_2_O_3_ precipitates enriched in Ti. The reason why Y_2_TiO_5_ precipitates were not observed is not clear, although it may also have to do with the availability of Ti in the matrix and the resulting Ti/Y ratio. Y and Ti were implanted in similar and relatively important concentrations (around 5–6 at.% at peak concentration), but the surface, 50 nm from the peak concentrations of the implanted elements, is a strong trap for the migrating species under annealing at 1100 °C, and Ti was shown to be drained towards the surface stronger than Y after annealing at 1100 °C in single Y and Ti-implanted specimens [32]. A lack of oxygen, also likely to be attracted to the surface during annealing, could also contribute to the formation of Y_2_O_3_ rather than Y_2_TiO_5_ precipitates. A quantitative compositional analysis of the precipitates would certainly enable us to elucidate the question, but it is unfortunately very hard to achieve with the present specimens, and beyond the scope of this paper. We may, however, note that pyrochlore precipitates are observed together with yttria precipitates in some ODS steels [34,53], but in this case, yttria precipitates may result from insufficiently mechanically processed Y_2_O_3_ powder. 

The results reported in Section 3.3.1 show that (Y,Ti)-rich oxide clusters can already be observed after annealing at a temperature of 800 °C in specimens implanted in the sequential order Y ➔ Ti ➔ O. As mentioned earlier, whilst the migration of Ti is triggered at an annealing temperature close to 800 °C in the case of Ti ➔ O implantation [24,25], making Ti atoms available for absorption by the Cr_2_O_3_ synthesized precipitates, neither Y nor Ti is visible in observable precipitates after annealing at 800 °C in the Ti ➔ Y ➔ O-implanted specimens, leading to the idea that both of them are involved in immobile Y-Ti-O clusters in this specific case (possibly stabilized additionally by trapped vacancies [20]). A participation of Ti in complex defects that can potentially act on the kinetics of precipitation is supported by the well-known practice of Ti addition in order to reduce the size of precipitates in ODS production [46,54,55,56]. Even though this size reduction can be partly ascribed to the higher atomic density of pyrochlore as compared to cubic yttria, the difference in atomic density is only of ~30%, and the difference in volume occupied by nanoprecipitates with and without Ti can be far beyond that. For example, in the case of Fe-14Cr-Y ODS (0.3 wt% Y_2_O_3_) and Fe-14Cr-YTi ODS (0.3 Y_2_O_3_, 0.3 Ti) the reported ratio of average precipitate diameters is larger than two [54]. Thus, it is quite evident that Ti affects the cluster nucleation, either by inhibiting diffusion through the formation of immobile clusters or by acting as a multiplier of nucleation sites for oxide nanoprecipitates, or both. In this sense, a relatively recent APT and TEM study [57] showed, for example, in addition to the large oxide nanoprecipitates, the presence of clusters of relatively small sizes (2–6 nm): with Ti alloying in an Fe-13.5Cr ODS steel, the number of clusters that are enriched in O, Y, Cr, and Ti, grows significantly, while their size decreases. These results have to, however, be toned down considering the possible APT artefacts encountered in the study of oxide precipitates in metallic matrices [44,58], notably the trajectory aberrations around the oxide precipitates leading to incorporation of ions from the matrix inside the precipitate regions.

**Table 2:** The calculated binding energies between Y-V pair and Ti atoms in different configurations, as indicated in Figure 12. Density functional theory-based calculations performed on 128-atomic *bcc* iron lattice with VASP code [59] using PAW-PBE pseudopotentials [60] with an energy cut-off of 450 eV and a 4 × 4 × 4 Monkhorst–Pack *k*-point grid [61]. Positive sign of binding energy means energy gain from putting defects in close vicinity.

Ti Atom Position in Figure 12	Separation between Ti and the Nearest Site in Y-V Dumbbell	Binding Energy, *E_b_* (eV)
1	1 NN	0.20
2	1 NN	0.22
3	1 NN	−0.30
4	2 NN	−0.06

In the present results of triple-ion implantation, when Y is implanted first, the characteristics of the obtained precipitates are thus very similar to those in ODS steels undoped with Ti: Yttria precipitates are obtained with specific fine features recurrently observed in conventional ODS steels that are particularly well reproduced after annealing at 1100 °C. A Cr-rich shell [15,44,51], as well as an apparently cuboidal shape of yttria precipitates with the main facets parallel to {200} matrix planes [34,62] are, for example, evidenced. Note that the shape of precipitates could actually be more complex than cuboidal, as shown in a study that revealed (Y, Ti)-rich precipitates with similar morphology to ours when the matrix was oriented along a [001] zone axis, and which revealed a truncated rhombic dodecahedron shape defined by the {100} and {110} matrix planes with observations on other matrix orientations [63]. The precipitate facets along low index matrix planes have already been ascribed to diffusional processes, occurring to lower the surface energy of the precipitates [35], and which probably take place during the growth of the precipitates. Additionally, whatever precipitates were formed in our study, i.e., Cr_2_O_3_ or Y_2_O_3_, depending on the order in the sequence of implantations, orientation relationships are found between precipitate and matrix lattices, with {110} matrix planes, which are the planes of highest atomic density in the *bcc* structure, always observed to be parallel to precipitate planes. This is a highly likely indication of certain FeCr matrix restrictions imposed on the oxide cluster nucleation, which force alignment along {110} matrix planes to minimize the interfacial energy and the elastic distortions due to the precipitate–matrix lattice parameter mismatch.

To conclude, the different results obtained after successive ion implantations in two different sequential orders indicate that the kinetics of oxide precipitation in ion implantation experiments is not necessarily determined by the relative thermodynamic stability of precipitating phases but can be driven by the kinetics of chemical composition modification in the implanted samples already at the RT implantation stage, even though the outcome of this kinetics is not obvious immediately after implantation and is developed only after subsequent high-temperature treatment. A Ti-first implantation is shown to impede the formation of Y or (Y, Ti) oxides, which are usually observed in ODS materials [12,15] and should be the most favourable ones thermodynamically. The standard enthalpy of formation of corundum Cr_2_O_3_ (−1140 kJ/mol oxide, −2.35 eV/atom) is indeed higher than that of Y_2_O_3_ (−1905 kJ/mol oxide, −3.98 eV/atom), Y_2_Ti_2_O_7_ (−3874 kJ/mol oxide, −3.77 eV/atom), and Y_2_TiO_5_ (−2670 kJ/mol oxide–calculated value [33], −3.88 eV/atom) [43,64,65]—the negative sign for the formation enthalpies of stable compounds is adopted here in compliance with the standard practice. Cr_2_O_3_ should thus be more difficult to form in standard conditions than Y and (Y,Ti) oxides. However, the promotion of chromium reordering and Cr_2_O_3_ nucleation by the primary Ti implantation, in conjunction with the low mobility of yttrium in the FeCr matrix, results in the preferential precipitation of a thermodynamically less favourable phase.

## 5. Conclusions

Ion beam synthesis involving the implantation of Y, Ti, and O ions was successfully used to induce the formation of nano-oxide precipitates in an Fe-10%Cr matrix. The nature of the precipitated nano-oxides is found to be highly sensitive to the order in the sequence of metallic ion implantation. Therefore, the precipitation evidenced after subsequent thermal annealing is not necessarily driven by purely thermodynamic arguments but rather by complex kinetics aspects related to the interactions between implanted elements and defects in the matrix. These atomic-scale processes, determining the precipitate nucleation pathways, already operate during implantation at RT. The implanted and alloy elements are very likely to bind differently between themselves and to implantation-induced defects depending on the order in the sequence of ion implantation, which affects their diffusivity and reactivity in the nucleation process during subsequent annealing. When Ti is implanted first, elongated, rod-shaped precipitates with a corundum hexagonal Cr_2_O_3_ structure are observed. On the contrary, when Y is implanted first, cubic Y_2_O_3_ precipitates enriched in Ti are obtained with fine characteristics that are recurrently reported in conventionally produced ODS steels, e.g., a core/shell structure and an apparently cuboidal shape with facets parallel to {200} matrix planes are evidenced. The observation of pyrochlore Y_2_Ti_2_O_7_ in surface pockets suggests that the Y_2_O_3_ structure of the embedded precipitates deeper in the matrix results from Ti depletion in the bulk of the matrix. Moreover, independently of the nature of the synthesized precipitates, the systematic involvement of the dense {110} matrix planes in the orientation relationships highlights a matrix restriction effect in the early stages of precipitation, with precipitate nucleation likely forced along {110} matrix planes to lower the interfacial energy and elastic distortions. The reproduction of the characteristic features of ODS nanoprecipitates shows that IBS is a powerful tool to study the formation mechanisms of oxide nanoprecipitates in ODS materials, and it also opens possibilities of tuning the precipitate distribution by changing the nature of the implanted ion and implantation parameters. 

## Figures and Tables

**Table 1:** Summary of ion beam synthesis conditions and corresponding observations.

Ion Implantation Sequence	Annealing Temperature	Observations
Ti ➔ Y ➔ O	800 °C	Cr_2_O_3_ rod-shaped precipitates
Y ➔ Ti ➔ O	800 °C	Y, Ti, O-rich precipitates
	1100 °C	Ti-enriched Y_2_O_3_ cuboidal precipitates with a Cr-enriched shell + Y_2_Ti_2_O_7_ surface pockets

## Data Availability

The data presented in this study are available on request from the corresponding author (S.J.-L.).
